# A Novel Role for ATM in Regulating Proteasome-Mediated Protein Degradation through Suppression of the ISG15 Conjugation Pathway

**DOI:** 10.1371/journal.pone.0016422

**Published:** 2011-01-26

**Authors:** Laurence M. Wood, Surendran Sankar, Ryan E. Reed, Arthur L. Haas, Leroy F. Liu, Peter McKinnon, Shyamal D. Desai

**Affiliations:** 1 Department of Microbiology, University of Pennsylvania, Philadelphia, Pennsylvania, United States of America; 2 Department of Biochemistry and Molecular Biology, Louisiana State University of Health Sciences Center-School of Medicine, New Orleans, Louisiana, United States of America; 3 Department of Pharmacology, Robert Wood Johnson Medical School, University of Medicine and Dentistry of New Jersey, Piscataway, New Jersey, United States of America; 4 Department of Genetics and Tumor Cell Biology, St. Jude Children's Research Hospital, Memphis, Tennessee, United States of America; Karolinska Institutet, Sweden

## Abstract

*Ataxia Telangiectasia* (A-T) is an inherited immunodeficiency disorder wherein mutation of the ATM kinase is responsible for the A-T pathogenesis. Although the precise role of ATM in A-T pathogenesis is still unclear, its function in responding to DNA damage has been well established. Here we demonstrate that in addition to its role in DNA repair, ATM also regulates proteasome-mediated protein turnover through suppression of the ISG15 pathway. This conclusion is based on three major pieces of evidence: First, we demonstrate that proteasome-mediated protein degradation is impaired in A-T cells. Second, we show that the reduced protein turnover is causally linked to the elevated expression of the ubiquitin-like protein ISG15 in A-T cells. Third, we show that expression of the ISG15 is elevated in A-T cells derived from various A-T patients, as well as in brain tissues derived from the ATM knockout mice and A-T patients, suggesting that ATM negatively regulates the ISG15 pathway. Our current findings suggest for the first time that proteasome-mediated protein degradation is impaired in A-T cells due to elevated expression of the ISG15 conjugation pathway, which could contribute to progressive neurodegeneration in A-T patients.

## Introduction

*Ataxia Telangiectasia* (A-T) (Boder-Sedgwick/Louis-Bar syndrome) is an inherited immunodeficiency disorder with a prevalence rate of 1 in 30,000–100,000 births [1]–[3]. A-T patients are characterized by pronounced facial spider veins (telangiectsia), recurrent sinopulmonary infections, and an irregular gait (ataxia) that results from progressive neuronal dysfunction [4]. These clinical presentations, which are secondary to sensitivity to ionizing radiation and a marked predisposition to cancer, were explicated in 1995 by Savitsky *et al*. as an autosomal recessive mutation in the *Ataxia Telangiectasia Mutated* (*ATM*) gene. ATM is a 370 kDa nuclear Ser/Thr kinase that contributes to the regulation of p53, BRCA1, and Chk2 signaling pathways, amongst others. As such, it is required for progression through mitotic checkpoints, double strand DNA repair, telomere repair, apoptosis and meiosis [2]. The central role of ATM in cell regulation suggests that a loss of function mutations in the *ATM* gene should result in broadly pleiotropic effects.

ISG15 (**I**nterferon-**S**timulated **G**ene **15**) is a member of the UBL (ubiquitin-like protein) superfamily of proteins that includes Nedd8 and SUMO1, amongst others [5], [6]. ISG15 is conjugated to its target proteins (ISGylation) in an enzymatic cascade that involves specific E1, E2 and E3 enzymes [7], [8]. Its conjugating enzymes are also induced by type I interferons [9]–[13]. UBE1L and UbcH8 have been identified as the specific E1 and E2 enzymes for ISG15 conjugation respectively [9], [13], [14]. Although several E3s have been identified as possible ISG15 E3 ligases, the major E3 for ISG15 appears to be HERC5 [10], [11]. However, the partial loss of ISG15 conjugates in HERC5-ablated cells suggests that other ISG15 E3 ligases may contribute to ISG15 conjugation *in vivo*. ISG15 conjugation is reversible. UBP43 has been identified as an enzyme responsible for the deconjugation of ISG15 from target substrates [15].

ISG15 exists in both free [16] and target-conjugated forms [7]. Initial studies suggested that free ISG15 serves as an immunomodulatory cytokine secreted by a non-canonical mechanism in response to Type I interferon induction [17]. As an extracellular cytokine, purified ISG15 can activate natural killer and cytotoxic T-cells [16], stimulate IFN-γ production [16], induce dendritic cell maturation [18] and neutrophil recruitment [19], suggesting the likely role of ISG15 in immune surveillance. Several studies now demonstrate that intracellular free ISG15 and conjugated ISG15 play an important role in innate immunity against viral infection [20], suggesting a crucial role of ISG15 in well-established immunological effects of interferon [21].

Early studies to determine the function of ISG15 suggested that ISGylation may facilitate proteasomal degradation of selected proteins. One study in support of this hypothesis demonstrated that overexpression of UBE1L stimulates PML/RARα degradation in NB4 APL cells treated with retinoic acid [22]. However, more recent work has demonstrated that direct ISG15 conjugation to Serpin 2a, JAK, or STAT1 does not increase their respective rates of degradation [23], [24], suggesting that the role of ISGylation in PML-RARα degradation may be indirect. On the other hand, it is becoming increasingly clear that ISG15 exerts its biological effect by interfering with the ubiquitin pathway. ISG15 inhibits the ubiquitylation of Gag and Tsg101 which prevents their interaction and blocks retroviral replication and release [25]. In addition, ISG15 inhibits Nedd4 ubiquitin ligase and, consequently, the ubiquitylation of VP40 viral particles essential for budding of Ebola viruses [26], [27]. Furthermore, ISG15 inhibits ubiquitin-mediated degradation of IRF3, a transcription factor involved in the interferon response, and enhances innate antiviral immunity [28]. A possible mechanism for broader inhibition of ubiquitin-dependent targeting pathways has been suggested by UbcH8-ISG15 acting as an alternate substitute for ligases specific for the closely related ubiquitin-specific UbcH7 and the UbcH5 clade [29]. Indeed, several groups have now demonstrated that ISG15 inhibit polyubiquitylation by modulating the activities of selected ubiquitin E2 and E3 ligases [26], [27], [30]–[32]. In normal cells, the ISG15 pathway is not constitutively elevated. However, when aberrantly overexpressed, ISG15 could either compete with ubiquitin to bind to the ubiquitin E2/E3 ligases as proposed by Haas [29], or it could conjugate to and inhibit the activity of ubiquitin E2/E3 ligases as demonstrated with Nedd4 [26], [27], UbcH6 [30], and UbcH13 [31], [32]. Inhibition of the ubiquitin ligation is, therefore, expected to decrease protein polyubiquitylation and proteasome-mediated degradation of cellular proteins. Indeed, ISG15 inhibits bulk polyubiquitylation and the subsequent 26S proteasome-mediated degradation of target proteins in breast cancer cells [33]. In addition, elevated expression of ISG15 suppresses camptothecin-induced proteasome-mediated degradation of topoisomerase I in breast cancer cells [34], further supporting the antagonistic role of ISG15 in ubiquitin-mediated degradation of many endogenous cellular proteins.

ISG15 is elevated and conjugated to cellular proteins in A-T cells [35]. However, the functional significance of ATM in regulating (suppressing) ISG15 expression and ISGylation is unclear. Because ISG15 interferes with ubiquitylation in tumor cells [33], it prompted us to test the possibility that ATM may regulate the ubiquitin/26S proteasome pathway through suppression of ISG15. Our results show that cells with defective ATM exhibit reduced protein polyubiquitylation and turnover. The reduced protein turnover in A-T cells is associated with elevated expression of ISG15. In addition, specific knockdown of either ISG15 or UbcH8, ISG15-specific conjugating enzyme (E2) restores protein polyubiquitylation and turnover in A-T cells. Our results support a model in which ATM negatively regulates proteasome-mediated protein degradation through the ISG15 conjugation pathway. In support of such a model, we have also demonstrated that the ISG15 pathway is elevated not only in various A-T cell lines derived from A-T patients, but also in brain tissues of ATM knockout mice and A-T patients. As defective protein turnover in neurons has been a hallmark of many neurological diseases [36]–[41], our results point to the possibility for the first time that ISG15-mediated impairment of protein degradation in A-T neurons could contribute to the progressive neurodegeneration in A-T patients. The presence of Lys63-linked polyubiquitin and ISG15 inclusions/aggregates in the mid-brain sections obtained from A-T patients further strengthens this possibility.

## Results

### Protein polyubiquitylation and degradation is reduced in cells deficient in ATM

**Figure 1:**
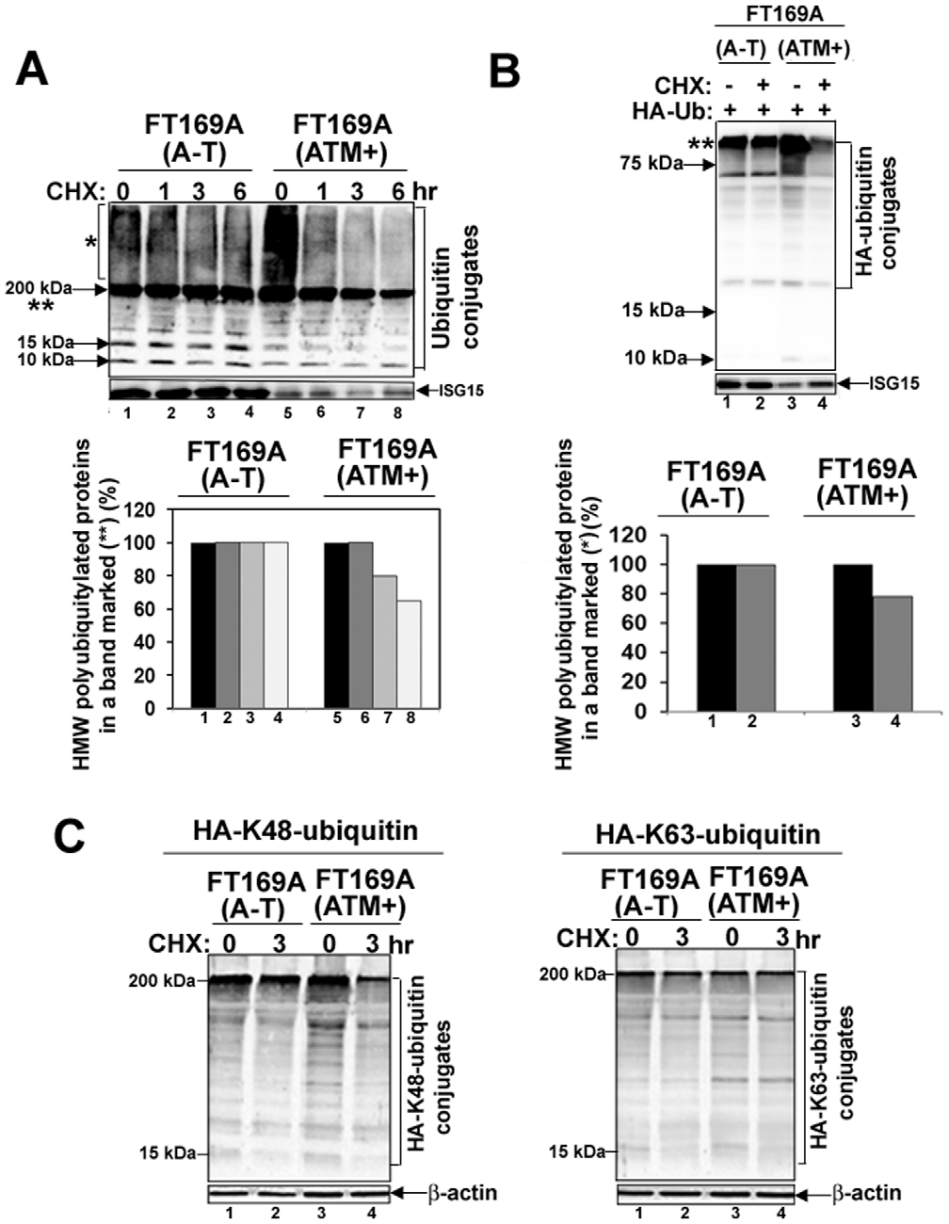
Protein turnover is reduced in A-T cells. A. FT169A (A-T) (lanes 1–4) and FT169A (ATM+) (lanes 5-8) cells were treated with the protein synthesis inhibitor CHX (10 µg/ml) for 0, 1, 3, and 6 hours. Cell lysates were analyzed using discontinuous (5%/15%) SDS-PAGE followed by immunoblotting with anti-ubiquitin antibody. The symbols * and ** mark the position of high-molecular-weight (HMW) polyubiquitylated proteins. Quantitation of the high-molecular-weight (HMW) polyubiquitylated proteins (shown as **) is shown in the bar graph. B. FT169A (A-T) (lanes 1 and 2) and FT169A (ATM+) (lanes 3 and 4) cells were transfected with HA-ubiquitin as described in Methods. Forty-eight hours post-transfection, cells were treated with the protein synthesis inhibitor CHX (marked on top of each lane) for 6 hours. Cell lysates were analyzed using 15% SDS-PAGE followed by immunoblotting with anti-HA antibody. The symbol ** marks the position of polyubiquitylated proteins (compressed due to the gel electrophoresis conditions). Quantitation of the high-molecular-weight (HMW) polyubiquitylated proteins (shown as **) is shown in the bar graph. C. FT169A (A-T) and FT169A (ATM+) cells were transfected with HA-Lys48-only (left panel) and Lys63-only (right panel) ubiquitin constructs. Thirty hours post-transfection, cells were treated with the protein synthesis inhibitor CHX (marked on the top of each lane) for 3 hours and then analyzed by immunoblotting with anti-HA antibodies as described above. All the experiments were repeated at least three times and the representative experiments are shown.

Accumulation of non-degraded proteins due to the lesions in the ubiquitin pathway has been speculated to contribute to the pathology of many neurodegenerative diseases [36]–[41]. *Ataxia Telangiectasia* (A-T) patients also show progressive neurodegeneration [42]. However, the reason for the progressive neurodegeneration in A-T is, so far, not known. To test whether like other neurodegenerating diseases, the defective ubiquitin-mediated degradation of cellular proteins contributes to neurodegeneration in A-T, we monitored the rate of degradation of overall cellular polyubiquitylated proteins in FT169A (A-T) (ATM null) and FT169A (ATM+) (ATM reconstituted FT169A) isogenic cells [43] using the protein synthesis inhibitor cycloheximide (CHX) [44]. As shown in Fig. 1A, the level of polyubiquitylated proteins (see protein species marked by * (smear of high molecular weight (HMW) ubiquitin-conjugated (polyubiquitylated) proteins and ** (high molecular weight polyubiquitylated proteins migrating as a compressed band) remained relatively unchanged in FT169A (A-T) cells up to six hours in the presence of CHX (compare lanes 1 and 4 and lower panel for quantification), suggesting minimal turnover of polyubiquitylated proteins in A-T cells. By contrast, the level of polyubiquitylated proteins (marked by* and **) was reduced by more than 30% within 6 hours in FT169A (ATM+) cells under the same conditions (Fig. 1A, compare lanes 5 and 8 and lower panel for the quantification). We also observed increased steady state level of the high molecular weight (HMW) ubiquitin-conjugated (polyubiquitylated) proteins (marked by *) in FT169A (ATM+) as compared to FT169A (A-T) cells (Fig. 1A, compare lanes 1 and 5) in Western analysis using anti-ubiquitin antibodies. The same membrane shown in Fig. 1A was stripped and re-probed with anti-ISG15 antibodies. The band intensities of the ISG15 protein remained same in FT169A (A-T) (lanes 1–4) and (ATM+) (lanes 5–8) cells (note that ISG15 protein levels are low in ATM+ as compared to A-T cells (see discussion below)) treated with CHX. These results revealed that targeted degradation of the polyubiquitylated proteins is specifically altered in A-T cells.

**Figure 2:**
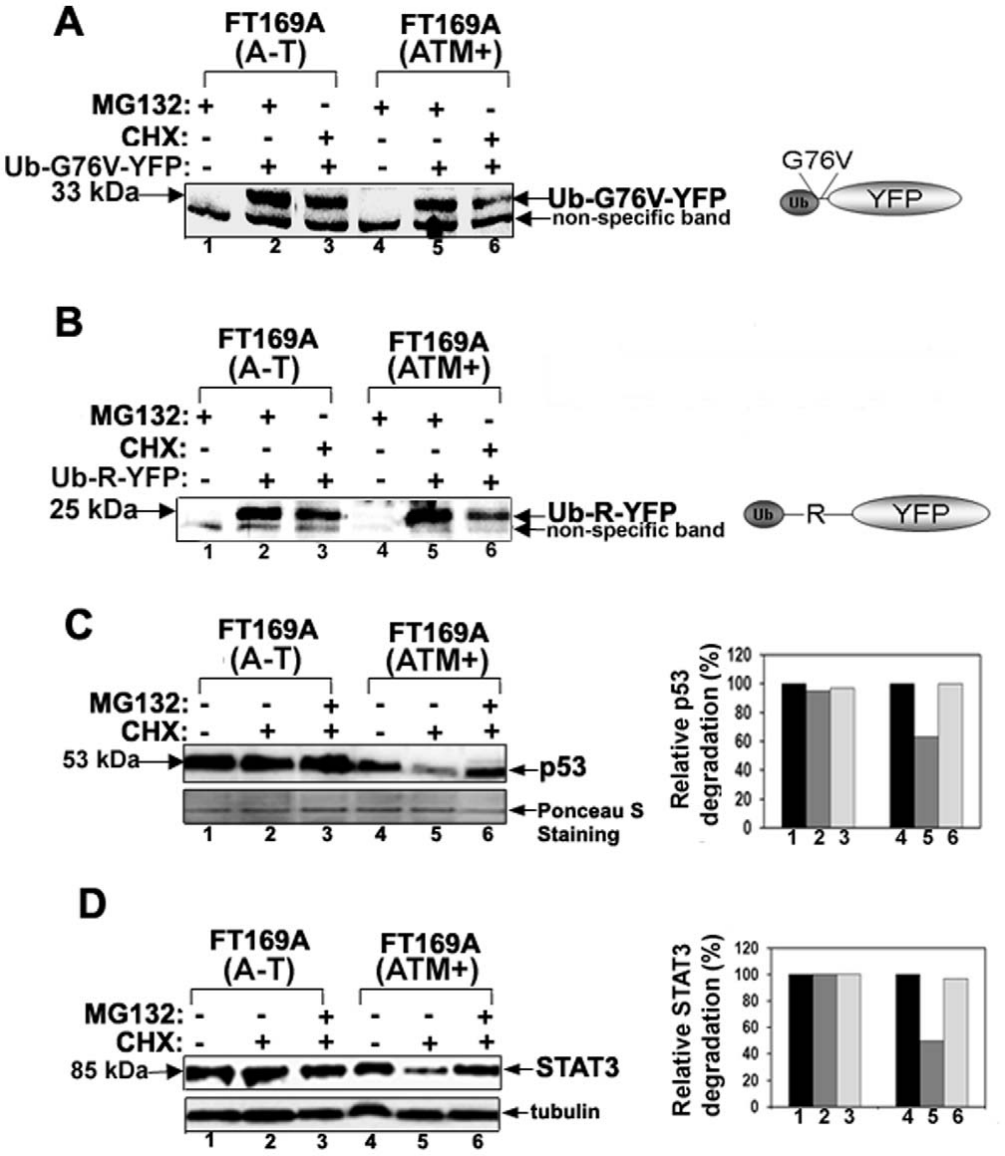
The 26S proteasome-mediated turnover of proteins is impaired in A-T cells. A and B. FT169A (A-T) and FT169A (ATM+) cells transfected with fluorescent reporter proteasome substrates (the ubiquitin fusion degradation substrate, Ub^G76V^ **-**YFP (panel A), and the N-end rule substrate, ubiquitin-arginine**-**YFP (Ub-R**-**YFP) (panel B) for 12 hours. Proteasome inhibitor MG132 (0.5 µM) was then added to the transfection medium and cells were allowed to grow for an additional 12 hours. After washing (to remove MG132), cells were treated with protein synthesis inhibitor CHX (10 µg/ml) for 3 hours. The fluorescent reporter levels were detected with GFP antibodies. C. FT169A (A-T) and FT169A (ATM+) cells were treated with the protein synthesis inhibitor CHX (10 µg/ml) in the presence (lanes 3 and 6) or absence (lanes 2 and 5) of the proteasome inhibitor MG132 (10 µM) for 6 hours. Cell lysates were analyzed by immunoblotting using an anti-p53 antibody (upper panel). The intensity of the p53 bands was measured using a Kodak Image station 2000R (BioRad). Results are shown in a bar graph (right panel). The filter used for immunoblotting was stained with Ponceau S to assure equal protein loading (lower panel). D. FT169A (A-T) (lanes 1-3) and FT169A (ATM+) (lanes 4–6) cells were treated with the protein synthesis inhibitor CHX (10 µg/ml) in the presence (lanes 3 and 6) or absence (lanes 2 and 5) of the proteasome inhibitor MG132 (10 µM) for 6 hours. Cell lysates were analyzed by immunoblotting using an anti-STAT3 antibody as described above. Intensity of the STAT3 band was measured using Kodak Image station 2000R (BioRad). Results are shown in a bar graph (right panel). The lower portion of the same membrane filter was immunostained with the anti-tubulin (lower panel) antibody. All of the experiments were repeated at least three times and the representative experiments are shown.

The ubiquitin antibody used in the above experiment is known to cross-react with free, but not conjugated, ISG15/UCRP [8]. In order to rule out the possibility that the polyubiquitylated proteins (see species marked by *) identified in Fig. 1A are not due to a cross-reaction with the ISG15 protein and/or other UBL-protein conjugates, HA-tagged ubiquitin cDNA was transfected into FT169A (A-T) and FT169A (ATM+) cells. The amount of polyubiquitylated proteins, and the rate of turnover of these polyubiquitylated proteins (see the HMW protein species marked by *) were then determined under the same conditions as in Fig. 1A, except that anti-HA, rather than an anti-ubiquitin antibody was used in immunoblotting. As shown in Fig. 1B, the amount of HMW HA-ubiquitin-conjugated (polyubiquitylated) proteins (marked by *) was elevated in FT169A (ATM+) as compared to FT169A (A-T) cells (Fig. 2B, compare lanes 1 and 4), consistent with results obtained by measuring the endogenous polyubiquitylated proteins in FT169A (A-T) and FT169A (ATM+) cells shown in Fig. 1A. The difference in the migration of polyubiquitylated proteins seen in Fig. 1A (migrating as a smear * and a compressed band **) and Fig. 1B (migrating as a compressed band **) is due to the different gel systems used in these experiments (5 and 15% discontinuous gel vs. 15% gel respectively). The turnover of HA-ubiquitin-conjugated proteins (species marked by **), measured in the presence of CHX (10 µg/ml) for 6 hours, was negligible in FT169A (A-T) cells (Fig. 1B, compare lanes 1 and 2 and lower panels for quantification). By contrast, about two-thirds of HA-ubiquitin-conjugated proteins were degraded in FT169A (ATM+) cells within 6 hours under the same conditions (Fig. 1B, compare lanes 4 and 5 and lower panels for quantification). The bar graph in the supplementary Fig. S1 shows average (+/- SEM) rate of degradation of the HA-polyubiquitylated proteins in FT169A (A-T) and (ATM+) cells from three independent experiments. The same membrane shown in Fig. 1B was stripped and re-probed with anti-ISG15 antibodies. The band intensities of the ISG15 protein remained unaltered in FT169A (A-T) (lanes 1–2) and (ATM+) (lanes 3–4) cells treated with CHX. These results revealed that targeted degradation of the HA-polyubiquitylated proteins is specifically altered in A-T cells. These results obtained with anti-HA-ubiquitin antibody are consistent with results obtained from the use of an anti-ubiquitin antibody (see Western blots (upper panels) and bar graphs showing quantitation of a 200 kDa band (**) (lower panels) comprised of polyubiquitylated proteins in Fig. 1A and B, and supplementary Fig. S1), suggesting that ATM regulates both the amount and the rate of degradation of polyubiquitylated proteins.

The ubiquitin sequence contains seven lysine residues (at positions 6, 11, 27, 29, 33, 48, and 63) and polyubiquitin chain assembly can occur at any of these lysine residues [45]. Lys48-linked polyubiquitylated proteins are targeted for destruction via the 26S proteasome [45]. On the other hand, a protein modification with Lys63-linked ubiquitin chains has been implicated in the non-proteolytic regulation of signaling pathways [45]. To test if the proteasome-mediated degradation of cellular proteins is impaired in A-T cells, we examined the steady state levels of HA-tagged Lys48- and Lys63-linked polyubiquitylated proteins in FT169A (A-T) and FT169A (ATM+) cells in the presence of CHX. For this purpose, the HA-Lys48-only and HA-Lys63-only constructs were transfected into FT169A (A-T) and FT169A (ATM+) cells. The amount of polyubiquitylated proteins and the rate of turnover of polyubiquitylated proteins (see the HMW protein species marked by *) were determined under the same conditions as in Fig. 1B using the anti-HA antibody in immunoblotting. As shown in Fig. 1C, the level of HA-Lys48-linked polyubiquitylated proteins remained relatively unchanged in A-T cells up to three hours in the presence of CHX (Fig. 1C, left panel, compare lanes 1 and 2), suggesting minimal turnover of Lys48-linked polyubiquitylated proteins in A-T cells. On the other hand, the cellular pool of Lys48-linked polyubiquitylated proteins was reduced by more than 70% within 3 hours in FT169A (ATM+) cells under the same conditions (Fig. 1C, left panel, compare lanes 3 and 4). By contrast, the levels of non-proteolytic HA-Lys63-linked polyubiquitylated proteins remained unchanged in both FT169A (A-T) and FT169A (ATM+) cells treated with CHX for 3 hours (Fig. 1C, right panel, compare lanes 1–4). These results suggest that targeted proteasome-mediated degradation of polyubiquitylated proteins is impaired in A-T cells.

To further determine whether proteasome-mediated degradation of cellular proteins are regulated by ATM, the steady state levels of two fluorescent reporter proteasome substrates (the N-end rule substrate, ubiquitin-arginine**-**YFP (Ub-R**-**YFP), and the ubiquitin fusion degradation substrate, Ub^G76V^ **-**YFP (gift from Dr. Nico Dantuma, Karolinska Institutet, Stockholm, Sweden [46]), were measured in FT169A (A-T) and FT169A (ATM+) cells in the presence of CHX. Cells expressing these reporter substrates are known to respond to functional impairment of the ubiquitin/proteasome pathway by accumulation of the readily detectable fluorescent reporter substrate [46]. Since these fluorescent substrates are short lived and are degraded rapidly by the proteasome *in vivo*, we pretreated cells expressing reporter YFP-substrates with the reversible proteasome inhibitor MG132 to enhance their accumulation. After 24 hours, cells were washed to remove MG132-mediated block in proteasome inhibition. The fate of these accumulated YFP-substrates was then monitored in the presence of CHX and in the absence of MG132, and Western blotting using anti-GFP antibodies (YFP differs from GFP due to a mutation at T203Y. Antibodies raised against full-length GFP can therefore detect YFP protein). As shown in Fig. 2A and B (lanes 2 and 3), little turnover of both Ub^G76V^-YFP and Ub-R-YFP was observed in FT169A (A-T) cells in the presence of CHX for up to three hours. By contrast, both of these YFP-substrates were rapidly degraded within 3 hours of CHX treatment in FT169A (ATM+) cells (Fig. 2A and B, lanes 5 and 6). Turnover of non-specific band remained unaltered under the same conditions in both of the cases and serves as an internal control. The bar graph in the supplementary Fig. S2 A shows average (+/- SEM) rate of degradation of the Ub-R-YFP and Ub-G76V-YFP proteins in FT169A (A-T) and (ATM+) cells from three independent experiments. These results suggest that targeted proteasome-mediated degradation of the proteasome substrates, in this case the artificial proteasome substrates, is impaired in A-T cells.

Both p53 and STAT3 are known targets of the ubiquitin/26S proteasome pathway [47], [48]. To determine whether steady state level of these proteins is regulated by ATM, turnover of both p53 and STAT3 were measured. As shown in Fig. 2C (upper panel, lanes 1 and 2), little turnover of p53 was observed in FT169A (A-T) cells in the presence of CHX for up to six hours. By contrast, p53 protein was rapidly degraded within 6 hours of CHX treatment in FT169A (ATM+) cells (Fig. 2C, lanes 4 and 5, and bar graph for p53 band quantization). The turnover of p53 in the presence of CHX was blocked by the proteasome inhibitor MG132 (10 µM), suggesting that p53 turnover was mediated by the 26S proteasome (Fig. 2C, compare lanes 5 and 6) in ATM+ cells. Similar results were obtained with STAT3 (see Fig. 2D and bar graph for STAT3 quantization). The bar graph in the supplementary Fig. S2B shows average (+/- SEM) rate of degradation of p53 and STAT3 proteins in FT169A (A-T) and (ATM+) cells from three independent experiments. Furthermore, we have previously demonstrated that CPT-induced topoisomerase I degradation via ubiquitin-26S proteasome is blocked in FT169A (A-T) cells but not in FT169A (ATM+) cells [34]. Together, these results suggest that ubiquitin/26S proteasome pathway is impaired in A-T cells. This is the first study indicating that ubiquitin/26S proteasome pathway is impaired in A-T cells.

### ATM negatively regulates the ISG15 pathway

**Figure 3:**
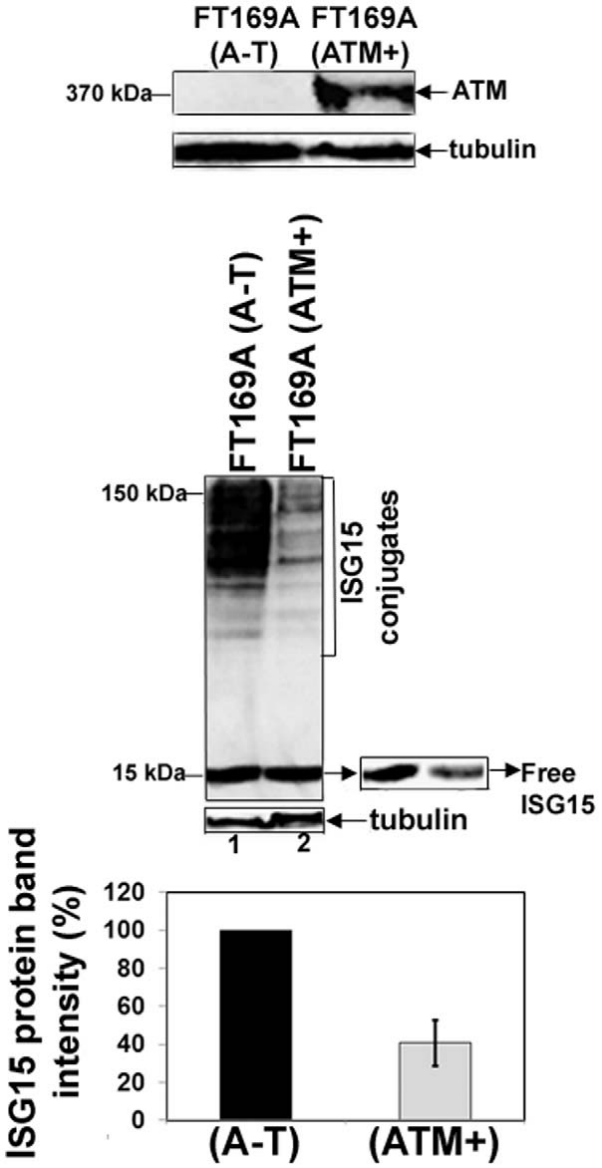
ATM negatively regulates the ISG15 pathway. Extracts of FT169A (A-T) and FT169A (ATM+) cells were analyzed by 5% (top panel) or 15% (middle panel) SDS-PAGE, followed by immunoblotting using either anti-ATM (top panel) or anti-ISG15 antibody (middle panel). The same membrane shown in the second panel was stripped and re-probed with anti-tubulin antibody to assure equal protein loading. Average band intensity of the free ISG15 protein (error bar represents SEM) from three independent experiments was quantified using Kodak Image Station 2000R and the results are shown in the bar graph.

Previous studies have shown that ISG15 is increased in A-T lymphoblasts [35] as well as in HeLa cells transfected with ATM siRNA [49]. Overexpression of ISG15 in tumor cells has been linked to reduced protein polyubiquitylation and turnover [33]. To determine whether overexpression of the ISG15 pathway is responsible for reduced protein polyubiquitylation in FT169A (A-T) cells (Figs. 1 and 2), we measured the levels of ISG15 and its conjugates in ATM null FT169A (A-T) and ATM-reconstituted FT169A (ATM+) cells using anti-ISG15 antibodies by Western analysis. As shown in the top panel of Fig. 3, no detectable ATM protein is present in FT169A (A-T) fibroblast cells. By contrast, ATM protein is readily detected in their corresponding wild type cells (i.e. FT169A (ATM+) cells). The levels of both free ISG15 and ISG15 conjugates were significantly higher in FT169A (A-T) cells than in their corresponding wild type FT169A (ATM+) cells (Fig. 3, middle panel). The bar graph in the supplementary Fig. S3 shows average (+/- SEM) band intensities of free ISG15 proteins in FT169A (A-T) and (ATM+) cells from three independent experiments. Our results using A-T fibroblast cells are thus consistent with the previous studies that, ISG15 is overexpressed in A-T lymphoblast cells [35]. These results, together with results shown in Fig. 1 and 2, suggest that like in tumor cells, overexpression of the ISG15 pathway result in reduced protein polyubiquitylation and turnover of cellular proteins in A-T cells.

### siRNA-mediated knockdown of ISG15 and UbcH8 increases protein polyubiquitylation and degradation in A-T cells

**Figure 4:**
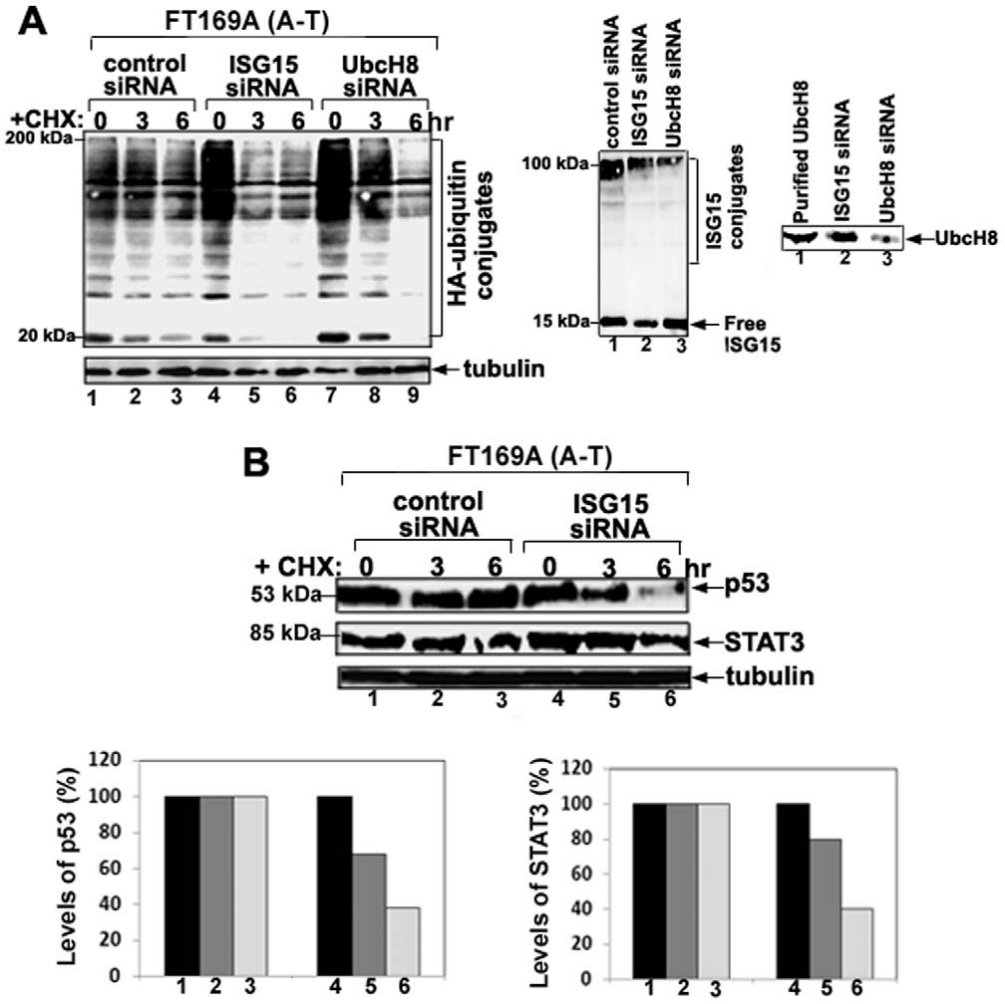
siRNA-mediated knockdown of ISG15 and UbcH8 increases protein polyubiquitylation and degradation in A-T cells. A. FT169A (A-T) cells were treated with either control (lanes 1–3), ISG15 (lanes 4–6) or UbcH8 (lanes 7–9) siRNAs for 72 hours followed by transfection with an HA-ubiquitin expression vector for 24 hours. Cells were treated with protein synthesis inhibitor CHX (10 µg/ml) for various times (lanes 2, 3, 5, 6, 8 and 9). Cells were then lysed with 2x SDS gel sample buffer. Cell lysates were then analyzed by immunoblotting using anti-HA antibody (first upper panel). The same membrane shown in the first upper panel was stripped and re-probed with anti-tubulin antibody to assure equal protein loading (first lower panel). The same samples shown in lanes 1, 4, and 7 were reloaded on a separate gel (15%), followed by immmunoblotting using an anti-ISG15 antibody (middle panel). The same samples shown in lanes 1 and 7 along with purified UbcH8 enzyme were reloaded on a separate gel (15%), followed by immmunoblotting using the anti-UbcH8 antibody (right panel). B. FT169A (A-T) cells were transfected with ISG15 siRNA for 72 hours. Cells were then treated with the protein synthesis inhibitor CHX (10 µg/ml) for 3 and 6 hours. Cell lysates were then analyzed by immunoblotting using anti-p53 (top panel), anti-STAT3 (middle panel) or anti-tubulin (lower panel) antibodies. The p53 and STAT3 bands shown in the first and second panels were quantified using the Kodak Image Station 2000R (see respective bar graphs). All the experiments were repeated at least three times and the representative experiments are shown.

To further determine whether overexpression of ISG15 and its conjugates in A-T cells are responsible for reduced protein polyubiquitylation and turnover, ISG15 and UbcH8 (the cognate E2 for ISG15 conjugation) siRNAs were employed to knockdown the expression of ISG15 and ISG15 conjugates, respectively, in FT169A (A-T) cells. Seventy-two hours after siRNA transfections, cells were further transfected with HA-ubiquitin cDNA for 24 hours. ISG15 siRNA significantly reduced ISG15 expression (70% decrease in the ISG15 band intensity) as revealed by immunoblotting using anti-ISG15 antibody (Fig. 4A, middle panel, compare lanes 1 and 2). UbcH8 siRNA, on the other hand, significantly reduced the amount of ISG15-protein conjugates without affecting the expression level of free ISG15, as revealed by immunoblotting using anti-ISG15 antibody (Fig. 4A, second panel, compare lanes 2 and 3). Western blotting analysis of FT169A (A-T) cells transfected with UbcH8 siRNA showed that the expression level of UbcH8 was reduced by 70% (as judged by the decrease in the UbcH8 band intensity) as compared to FT169A (A-T) cells transfected with control siRNA (Fig. 4A, third panel). By contrast, under the same conditions, the amount of HA-ubiquitylated HMW proteins (reflecting polyubiquitylated proteins), revealed by immunoblotting with anti-HA antibodies, was greatly increased in cells treated with either ISG15 or UbcH8 siRNA than in cells treated with control siRNA (Fig. 4A, first panel, compare lane 1 with lanes 4 and 7). The turnover of polyubiquitylated proteins was then measured in the presence of CHX (see Fig. 4A). As shown in the left panel of Figure 4A, the turnover of HA-ubiquitin-conjugated proteins was negligible in FT169A (A-T) cells (overexpressing ISG15) treated with CHX (10 µg/ml) for 6 hours (compare lanes 1 and 3). By contrast, about two thirds of HA-ubiquitin-conjugated proteins were degraded in FT169A (A-T) cells transfected with either ISG15-specific (Fig. 4A, first panel, compare lanes 4 and 6) or UbcH8-specific siRNA (Fig. 4A, first panel, compare lanes 7 and 9) within 6 hours under the same conditions. The same membrane filter as shown in Fig. 4A was stripped and re-probed with anti-tubulin to assure equal protein loading (Fig. 4A, lower panel). We also show that the turnover of p53 and STAT3, which is reduced in FT169A (A-T) cells transfected with control siRNA, is restored in FT169A (A-T) cells transfected with ISG15 siRNA (Fig. 4B first and second panel, and bar graph for quantitation). These results suggest, in part, that protein ISGylation results in reduced protein polyubiquitylation and turnover of cellular proteins in A-T cells. However, we cannot rule out the possibility that the free ISG15 pool also plays an independent role in regulating protein polyubiquitylation and turnover in A-T cells.

### Expression of ISG15 and its conjugates is elevated in cells deficient in ATM

**Figure 5:**
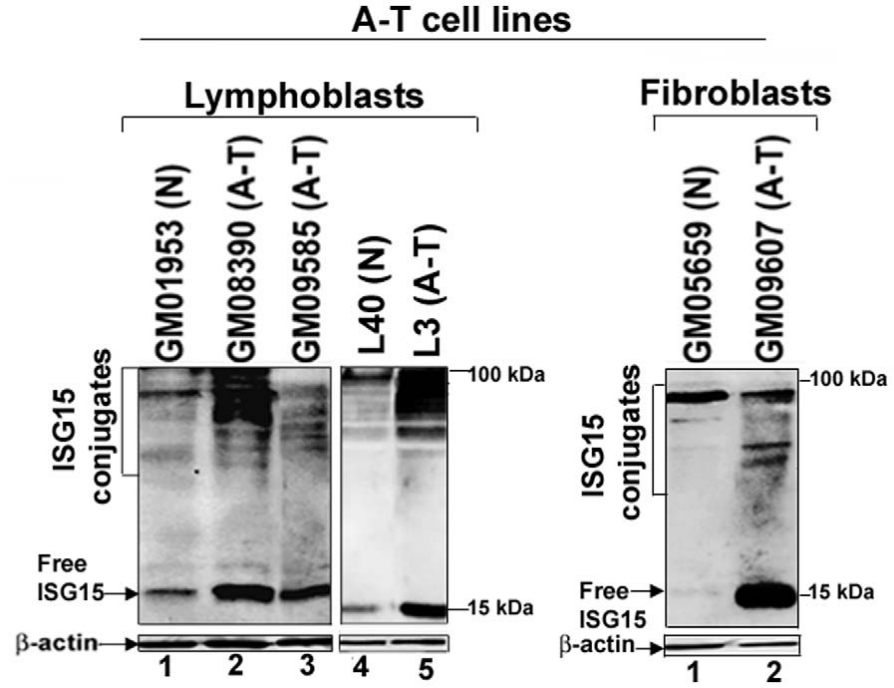
Elevated expression of ISG15 and its conjugates in A-T cells. Normal (N) and Ataxia Telangiectasia (A-T) lymphoblast (left panel) and fibroblast (right panel) cells were analyzed by 15% SDS-PAGE, followed by immunoblotting using anti-ISG15 antibody (upper panels). The same membrane shown in the upper panels was stripped and re-probed using anti-β-actin antibody (lower panels). The experiment was repeated at least three times and the representative experiment is shown.

We also measured the levels of ISG15 and its conjugates in several other lymphoblast and fibroblast cell lines derived from A-T patients (A-T) and normal individuals (N). As shown in Fig. 5A, the levels of ISG15 and its conjugates as measured by immunoblotting using anti-ISG15 antibodies were higher in A-T lymphoblast (left panel, lanes 2, 3 and 5) and fibroblast (right panel, lane 2) cells. On the other hand, very little ISG15 expression (free and conjugated form) was seen in both lymphoblast and fibroblast cells derived from normal individual (left panel, lanes 1 and 4, and right panel, lane 1). These results, together with the results shown in Fig. 3, strongly suggest that ATM negatively regulates the expression of ISG15 and its conjugates.

### Expression of ISG15 and its conjugates is elevated in brains of ATM knockout mice and A-T human patients

**Figure 6:**
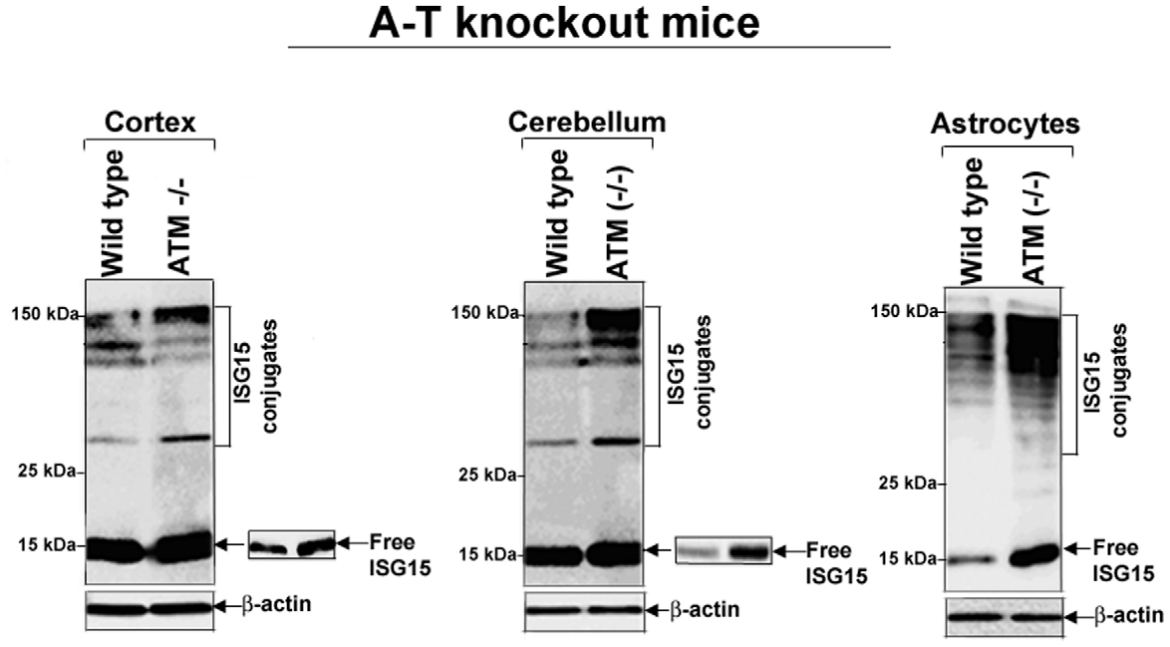
ISG15 and its conjugates are elevated in brain tissues obtained from ATM knockout mice. Lysates from cortex (left panel) and cerebellum (middle panel) tissues, as well as primary cortical astrocytes (right panel), were immunoblotted using anti-ISG15 antibodies as described in Material and Methods. All membrane filters were immunostained with anti-tubulin antibody (lower panels). The brain tissue lysates of two animals were pooled and loaded on SDS-PAGE. The experiment was repeated two times with reproducible results.

Our results shown in Figs 1, 2, 3, 4 suggest the possibility that the ISG15-mediated impairment of protein degradation in A-T neurons could be the basis of the progressive neurodegeneration in A-T patients. To test whether ISG15 expression is also elevated *in vivo*, we first assessed the expression of ISG15 and its conjugates in various regions of brain tissues obtained from wild type and ATM knockout mice. The levels of free ISG15 (see inserts showing lower exposure) and its conjugates were increased in the cortex (Fig. 6, first panel) and cerebellum (Fig. 6, second panel) parts of the brains isolated from ATM knockout as compared to wild-type mice. In addition, ATM knockout astrocytes exhibited striking increase in ISG15 and its conjugates than astrocytes derived from wild-type mice (Fig. 6, third panel).

**Figure 7:**
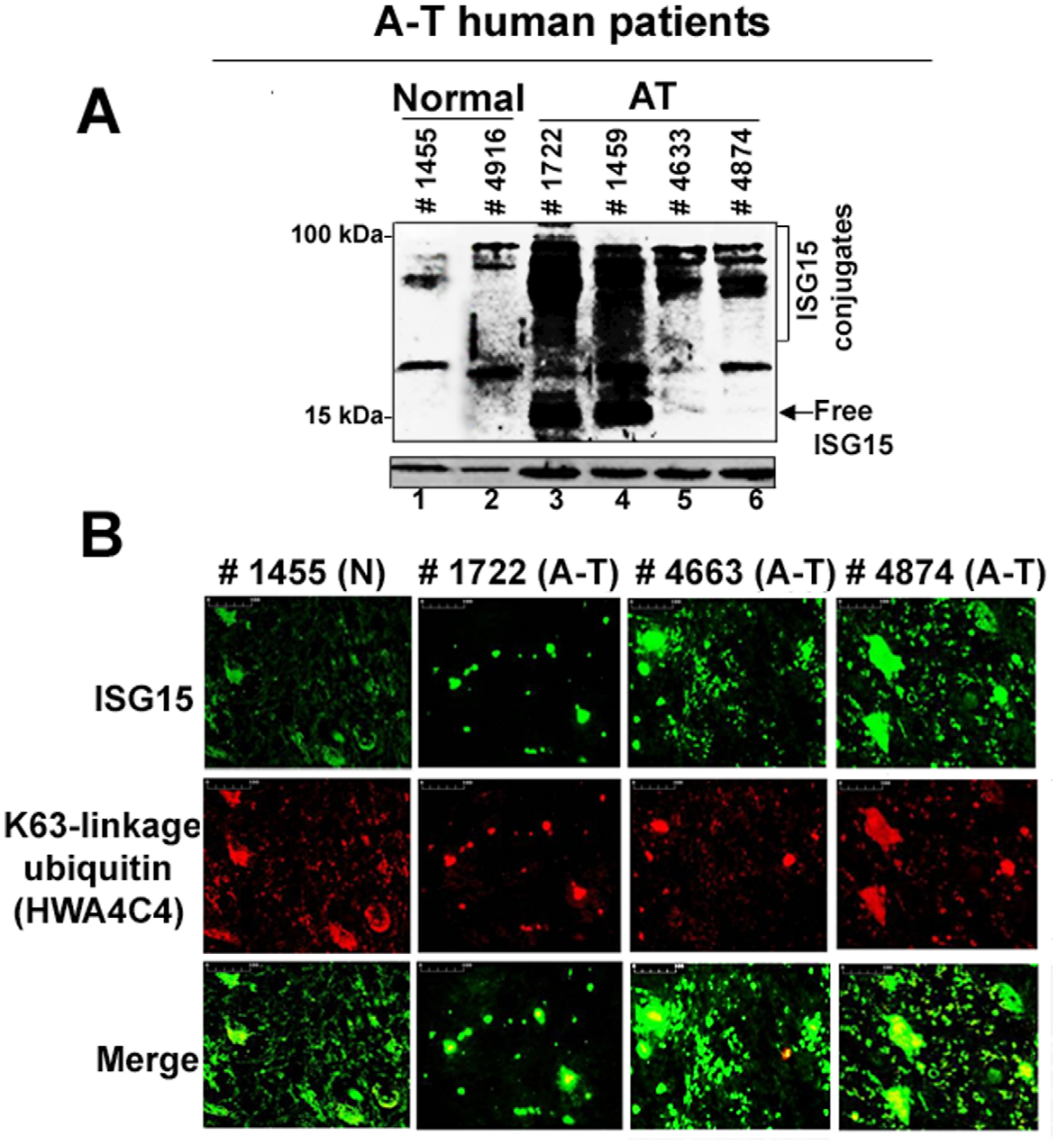
Elevated expression of ISG15 and its conjugates in mid-brain tissues obtained from human A-T patients. A. Frozen mid-brain postmortem tissues from two normal individual (UMB# 1455 and 4916) and four A-T patients (UMB #s 1722, 1459, 4663 and 4874) were weighed and sonicated in a SDS sample buffer. Sonicated samples were immediately boiled for 10 min at 100°C and centrifuged at 13,000×g for 10 min. Cleared supernatants were analyzed using anti-ISG15 antibodies. As a loading control, lysates were also immunoblotted aganist β-actin. B. The deparaffinized human brain tissue sections from the normal subject (UMB# 1455) and A-T patients (UMB# 1722, 4663) described in A. were double stained with anti-ISG15 (polyclonal) and anti-K63-linkage specific polyubiquitin (monoclonal) (1∶100) antibodies. After washing with PBS, sections were stained with Alexa Fluor 488 goat anti-rabbit IgG secondary antibody to detect ISG15 (green) and goat polyclonal secondary antibody to mouse IgG (Cy5®) to detect Lys63-linked polyubiquitin conjugated proteins (red). Sections were mounted in gold antifade mounting medium and examined using Nikon E600 epifluorescence microscope (Nikon) (20× magnification, scale bar, 100 um). One slide each of the deparaffinized human brain tissue sections of A-T patients and normal individuals (obtained from the NICHD Brain and Tissue Bank for Developmental Disorders at the University of Maryland) was used in the experiment. Arrows indicate ubiquitin/ISG15 double-positive inclusions in the A-T brain sections.

To further examine if ISG15 expression is elevated *in vivo*, we assessed mid-brain regions (specifically containing substantia nigra) obtained postmortem from four different A-T human patients (with confirmed A-T disease (UMB#s 1722, 1459, 4663, and 4874)) and two control individuals (without any disease (UMB#s 1455 and 4916)) for ISG15 expression by Western blotting using anti-ISG15 antibodies. As shown in Fig. 7 A, ISG15 and its conjugates were highly elevated in two A-T patients (lanes 3 and 4), and moderately elevated in two other A-T patients (lanes 5 and 6) tested. On the other hand, ISG15 expression was modest in brain tissue obtained from normal individuals (lane 1 and 2).

We also performed double immunofluorescence analysis on the mid-brain tissue sections (containing specifically substantia nigra) obtained from a normal individual (UMB#1455) and A-T patients (UMB# 1722, #4663, and #4874) shown in Fig. 7A, using ISG15 (green) and Lys63-linkage-specific polyubiquitin (red) antibodies (Fig. 7B). We used Lys63-linkage-specific antibodies instead of ubiquitin in this experiment for the following two reasons: i) ubiquitin antibody cross-reacts with free ISG15 [8]; and ii) presence of the Lys63-linked polyubiquitylated proteins in the inclusion bodies/aggregates has been documented in various neurological disorders (Paine et al., 2008). As shown in Fig. 7B, the dramatic increase in both ubiquitin/ISG15 double-positive inclusions (see arrows in merged images) was found in the mid-brain sections obtained from all three A-T patients tested. In contrast, no such inclusions were found in brain sections of the normal individual. We also performed an immunofluorescence study on the mid-brain section obtained from another normal subject (UMB# 4669); consistent with the results shown in Fig. 7B (UMB# 1455), no ISG15 containing inclusion bodies were found in the brain sections obtained from this normal individual using ISG15-specific antibodies (results not shown). The presence of ISG15/Lys63-linkage specific polyubiquitin containing inclusion bodies in the A-T patient's brain sections further suggests the involvement of a defective ubiquitin-proteasome system in A-T neurodegeneration.

## Discussion

*Ataxia-telangiectasia* is a childhood disease with diverse clinical manifestations that results from inactivation of the ATM kinase [1], [3]. ATM is known to play a key regulatory role in DNA repair by transducing the DNA damage signals to cell cycle checkpoints and apoptosis [1], [3]. However, it is not clear whether the role of ATM in DNA repair is solely responsible for A-T pathogenesis. In the current study, we have demonstrated that targeted proteasome-mediated degradation is impaired in A-T cells. In addition, reduced protein turnover in A-T cells is associated with elevated expression of ISG15, an ubiquitin-like protein shown to antagonize the ubiquitin pathway [33]. Furthermore, ATM acts as a suppresser of the ISG15 pathway. Our results therefore assign a novel functional role for ATM in protein turnover through suppression of constitutively activated ISG15 pathway in normal cells. Due to the inactivation of ATM kinase ISG15 pathway is elevated which, in turn, inhibits ubiquitin-mediated protein turnover in A-T cells.

Defects in the ubiquitin pathway resulting in the accumulation of non-degraded proteins in neurons have been speculated to contribute to the neurodegenerative pathology in other neurological diseases/proteinopathies [36]–[41], [50], [51]. A-T patients also exhibit progressive neurodegeneration [42]. However, the cause of neurodegeneration in A-T remains unclear. It seems reasonable to speculate that accumulation of non-degraded proteins, as would be predicted from our results, may contribute to progressive neurodegeneration in A-T patients. Indeed, a couple of reports in the literature support such a possibility. For example, immunopositive bodies for alpha-synuclein (due to accumulation of non-degraded alpha-synuclein) were observed in the substantia nigra of ATM knockout mice [42]. In addition, Lewy bodies (commonly found in Parkinson's patients due to accumulation of non-degraded ubiquitylated proteins) in the substantia nigra were found in a patient with Ataxia-Telangiectasia [52]. In the current study, we show that ISG15 is elevated in A-T astroglial cells and brain tissue obtained from ATM knockout mice and patients (Figure 6 and 7). In addition, we also show the presence of ubiquitin/ISG15 double-positive inclusions in the brain sections obtained from A-T patients (Fig. 7). Whether or not the elevated expression of ISG15 impairs protein turnover and, consequently, leads to the accumulation of ubiquitin/ISG15 inclusions in A-T patients is unclear.

Defective DNA repair due to the mutations in ATM protein is also implicated in A-T neurodegeneration [53]. Although our current study points toward the possibility that the defective protein turnover may contribute to the A-T neurodegeneration, it does not rule out the possibility that the defective DNA repair may also contribute to A-T pathology (neurodegeneration). In fact, several recent studies have demonstrated that DNA repair is regulated by ubiquitylation [54]. It is possible that both the impaired DNA repair and targeted protein degradation (as demonstrated in the current study), both caused due to the defective ATM, may contribute to A-T neurodegeneration. Studies are underway to establish the significance of ISG15-mediated impairment of protein polyubiquitylation and turnover in A-T pathogenesis (e.g. neurodegeneration) in our laboratory using ATM knockout mice.

At present, it is unclear how ATM regulates the expression of ISG15. One possibility is that ATM may negatively regulate ISG15 expression by inhibiting expression of type I IFNs. Concurrently, we have shown that A-T lymphoblastic L3 cells secrete high levels of type I IFNα than ATM+ L40 cells (data not shown). Our observation is consistent with the report that IRF1 (Interferon Regulatory Factor 1) is stabilized in ATM-deficient cells [55]. In addition, IFNβ pathway is activated in A-T cells [35]. Constitutive activation of NFκB has been suggested to be involved in IFNβ expression in A-T cells [35]. In contrast, other studies have shown that the aberrant activation of NFκB is causally related to the transformation process associated with SV40 large T antigen and is not due to the inactivation of ATM in A-T cells [56]. However, our results show that ISG15 is elevated in SV40 transformed A-T GM09607 (Fig. 3), as well as in non-transformed A-T GM01588 cells [35]. In addition, we have demonstrated that ISG15 expression is causally related to the ATM inactivation in A-T cells (Fig. 3). Further, our results in ATM knockout mice and A-T human patients (Figures 6 and 7), and HeLa cells expressing ATM siRNA (Chen et al., 2004) strongly support a model in which ablation of ATM kinase leads to the elevated expression of ISG15. Our studies thus have potential clinical applications for treating A-T patients. Development of small molecule inhibitors targeting ISG15 pathway could prevent proteinopathy associated with A-T and, consequently, neurodegeneration.

## Materials and Methods

### Ethic Statement

Human brain tissues and tissue sections were obtained from the NICHD Brain and Tissue Bank for Developmental Disorders at the University of Maryland (supported by NICHD contract # N01-HD-4-3368 and N01-HD-4-3383) under ethics protocols approved by the University of Maryland Institutional Review Board. The NICHD Brain and Tissue Bank for Developmental Disorders at the University of Maryland obtain informed consent from all participants or next of kin involved in donating tissue to the Brain and Tissue bank. Depending on the case/control, the consent may be written or verbal. Most cases are preregistered with the Brain and Tissue Bank (BTB) and written consent is obtained at that time. Next of kin are consented for cases that are not preregistered and for controls. Verbal consent is obtained when the person giving consent does not have access to a FAX machine or the ability to send documents by the internet, or because of time constraints. The verbal consent process is done by telephone using a script that is approved by both the University IRB and Maryland State Department of Mental Health and Hygiene. The verbal consent is confirmed by a witness who speaks with the person (next of kin) giving consent to tissue donation. The consent procedures have been approved by both the University of Maryland IRB and the Maryland State Department of Mental Health and Hygiene.

Animal study was carried out in strict accordance with the recommendations in the Guide for the Care and Use of Laboratory Animals of the National Institutes of Health. The protocol was approved by the Louisiana State University Health Sciences Center- NO Institutional Animal Care and Use Committee under its assurance (# A3094-0) with the Office of Laboratory Animal Welfare of the National Institutes of Health.

### Cells

Normal lymphoblast L40 and A-T lymphoblast L3 (ATM-) cells, as well as FT169A (ATM+) and FT169A (ATM-) fibroblast cells, were obtained from Dr. Y. Shiloh at Tel Aviv University, Ramat Aviv, Israel. FT169A (ATM-) cells were derived from FT169A cells (*ataxia telangiectasia* cells) by stable transfection with the expression vector alone [43]. FT169A (ATM+) cells were derived from FT169A cells by stable transfection with full-length ATM cDNA [43]. The lymphoblast and fibroblast cells (normal and A-T) were obtained from the American Type Culture Collection (ATCC), Manassas, VA. FT169A (ATM+) FT169A (ATM-) cells were cultured in DMEM supplemented with hygromycin B (50 µg/ml). L40 and L3 cells were cultured in RPMI medium. All other normal and A-T fibroblast cells were cultured in DMEM and lymphoblast cells were cultured in RPMI medium according to ATCC instructions.

### Human Tissues

#### Tissues

Frozen human mid-brain tissues containing specifically substantia nigra were obtained postmortem from patients with confirmed A-T disease and control individuals (without any known disease). *Tissue sections:* Slides with paraffin-embedded sections of the same midbrain tissues described above were used in immunoflorescence study.

### Immunoblotting and immunofluorescence analysis

#### Analysis of ISG15 in cultured cells

Cells (5×10^5^) were cultured in 35 mm tissue culture plates. Cells were then lysed using a SDS-PAGE sample buffer. Cell lysates were analyzed by SDS-PAGE in 15% (unless indicated otherwise) gel and immunoblotted according to the published procedure [57]. Cell lysates were analyzed by immunoblotting with anti-ISG15 (raised against human ISG15) [58], anti-ubiquitin (Sigma Chemicals), anti-HA (gift from Dr. Walworth at RWJMS/UMDNJ), anti-p53 (Santa Cruz), anti-STAT3 (Cell Signaling), and anti-GFP (Abcam) antibodies as indicated using the ECL Western procedure (Pierce) and the Kodak Image Station 2000R.

#### Analysis of ISG15 expression in brain tissues of *Atm* knockout mice

*Atm* knockout mice are described in [59]. Brain tissues were obtained from 3 week-old WT or *Atm* knockout littermates and stored in liquid nitrogen prior to processing. For detecting ISG15 and its conjugates, frozen tissues were weighed, cut into small pieces, and placed in test tubes containing SDS gel sample buffer. Tissue samples were then sonicated with a Tissue-Tearor (Biospec Products, Inc.). Sonicated samples were immediately boiled for 10 minutes at 100°C and then centrifuged at 13,000×*g* for 10 minutes. Cleared supernatants containing SDS-solubilized protein extracts were analyzed by SDS-PAGE in 15% gel and immunoblotted using anti-ISG15 raised against mouse ISG15 (a gift from Dr. Knobeloch, Institute of Molecular Pharmacology, Berlin, Germany) as described above.

#### Analysis of ISG15 expression in primary cortical astrocytes

Primary cortical astrocytes prepared from the brains of postnatal day-4 wild type and *Atm* knockout littermates were maintained as monolayers in DMEM/F12 (1∶1 mix) supplemented with a 15% fetal calf serum, 2 mM L-glutamine, 100 U/ml penicillin, 100 µg/ml streptomycin, and 10 ng/ml of mouse epidermal growth factor (Sigma, St Louis) and were used at passage 2. Cells were lysed using an SDS gel sample buffer. SDS-PAGE analysis and immunoblotting using mouse anti-ISG15 were carried out as described above.

#### Analysis of ISG15 expression in brain tissues of A-T patients by Western blotting

Frozen tissues were stored at −80°C until use. Tissue processing and ISG15 analysis in tissue lysates were carried out as described above.

#### Analysis of ISG15 expression in brain tissue sections by immunofluorescence staining

For double immunofluorescence, tissue sections were deparaffinized in xylene and incubated with the ISG15 (polyclonal) (1∶100) and polyubiquitin (K^63^-linkage-specific) (monoclonal HWA4C4 (Biomol)) primary antibodies (1∶100) and for 1 hour. After washing in PBS, sections were stained with Alexa Fluor 488 goat anti-rabbit IgG secondary antibody (Invitrogen) and goat polyclonal secondary antibody to mouse IgG (Cy5®) (Abcam). Sections were mounted in gold antifade mounting medium (Invitrogen) and examined using Nikon E600 epifluorescence microscope (Nikon) (20× magnification). All the operations were performed at room temperature.

### siRNA Knockdown of ISG15

A 21-nucleotide duplex siRNA targeting ISG15, and control siRNA were purchased from Dharmacon Research, Inc. The siRNA targeting ISG15 corresponds to region 232–250 (Accession# AY168648). The siRNA transfection protocol was followed with slight modifications [60]. FT169A (ATM-) cells were cultured to semi-confluency and transfected with ISG15 siRNA using Oligofectamine (Invitrogen). Seventy-two hours after siRNA transfection, cells were further transfected with HA-ubiquitin expression plasmid using PolyFect (Qiagen) for another 24 hours.

### siRNA knockdown of UbcH8

A 21-nucleotide duplex siRNA targeting UbcH8 siRNA was purchased from Dharmacon Research, Inc. The siRNA targeting UbcH8 corresponds to the region 237-255 (Accession# AF031141). The UbcH8 siRNA transfection, followed by HA-ubiquitin transfection, into FT169A cells was carried out as described above.

## Supporting Information

Figure S1 **The targeted degradation of ubiquitylated proteins is reduced in A-T cells.** FT169A (A-T) and FT169A cells were transfected with HA-ubiquitin as described in Methods. Forty-eight hours post-transfection, cells were treated with the protein synthesis inhibitor CHX for 6 hours and then analyzed by immunoblotting with anti-HA antibodies. The high molecular weight HA-polyubiquitylated proteins (in 200 kDa compressed band (see band marked as ** in Fig. 1B)) were detected with HA antibodies. Average rate of degradation of high molecular weight (HMW) HA-polyubiquitylated proteins (error bar represents S.E.M.) in FT169A (A-T) and FT169A (ATM+) cells measured using the Kodak image station 2000R from three independent experiments is shown in the bar graph.
(TIF)

Figure S2 **The 26S proteasome-mediated turnover of proteins is impaired in A-T cells.** A. FT169A (A-T) and FT169A (ATM+) cells transfected with fluorescent reporter proteasome substrates (the ubiquitin fusion degradation substrate, Ub^G76V^ **-**YFP, and the N-end rule substrate, ubiquitin-arginine**-**YFP (Ub-R**-**YFP) for 12 hours. Proteasome inhibitor MG132 (0.5 µM) was then added to the transfection medium and cells were allowed to grow for an additional 12 hours. After washing (to remove MG132), cells were treated with protein synthesis inhibitor CHX (10 µg/ml) for three hours. The fluorescent reporter levels were detected with GFP antibodies. Average rate of degradation of Ub-G76V-YFP and Ub-R-YFP proteins (error bar represents S.E.M.) in FT169A (A-T) and FT169A (ATM+) cells measured using the Kodak image station 2000R from three independent experiments is shown in the bar graph. B. FT169A (A-T) and FT169A (ATM+) cells were treated with the protein synthesis inhibitor CHX (10 µg/ml) for 6 hours. Cell lysates were analyzed by immunoblotting using an anti-p53 and/or STAT3 antibody. Average rate of degradation of p53 and STAT3 proteins (error bar represents S.E.M.) in FT169A (A-T) and FT169A (ATM+) cells measured using the Kodak image station 2000R from three independent experiments is shown in the bar graph.
(TIF)

Figure S3 **siRNA-mediated knockdown of ISG15 and UbcH8 increases degradation of polyubiquitylated proteins in A-T cells.** FT169A (A-T) cells were transfected with ISG15 or UbcH8 siRNA for 72 hours. Cells were then treated with the protein synthesis inhibitor CHX (10 µg/ml) for 3 and 6 hours. Cell lysates were then analyzed by immunoblotting using anti-HA antibodies. Average rate of degradation of HA-polyubiquitylated proteins (error bar represents S.E.M.) in ISG15 or UbcH8 siRNA treated FT169A (A-T) cells measured using the Kodak image station 2000R from three independent experiments is shown in the bar graph.
(TIF)
