# Supplementary figures and images for: A Novel Role for ATM in Regulating Proteasome-Mediated Protein Degradation through Suppression of the ISG15 Conjugation Pathway
Source: PLoS One. 2011 Jan 26;6(1):e16422. doi: 10.1371/journal.pone.0016422 (PMC3027683; doi:10.1371/journal.pone.0016422)

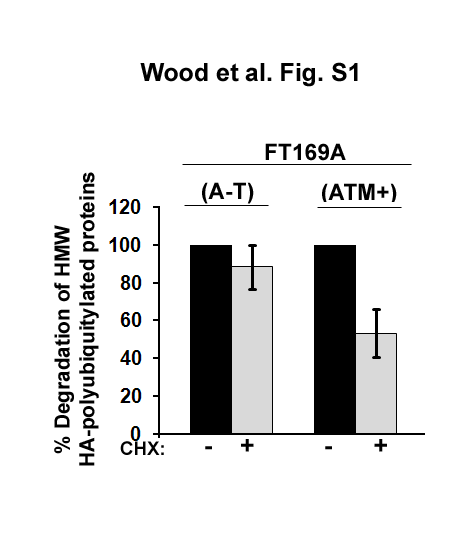

Supplement: Figure S1 — The targeted degradation of ubiquitylated proteins is reduced in A-T cells. FT169A (A-T) and FT169A cells were transfected with HA-ubiquitin as described in Methods. Forty-eight hours post-transfection, cells were treated with the protein synthesis inhibitor CHX for 6 hours and then analyzed by immunoblotting with anti-HA antibodies. The high molecular weight HA-polyubiquitylated proteins (in 200 kDa compressed band (see band marked as ** in Fig. 1B)) were detected with HA antibodies. Average rate of degradation of high molecular weight (HMW) HA-polyubiquitylated proteins (error bar represents S.E.M.) in FT169A (A-T) and FT169A (ATM+) cells measured using the Kodak image station 2000R from three independent experiments is shown in the bar graph. (TIF) [file pone.0016422.s001.tif]

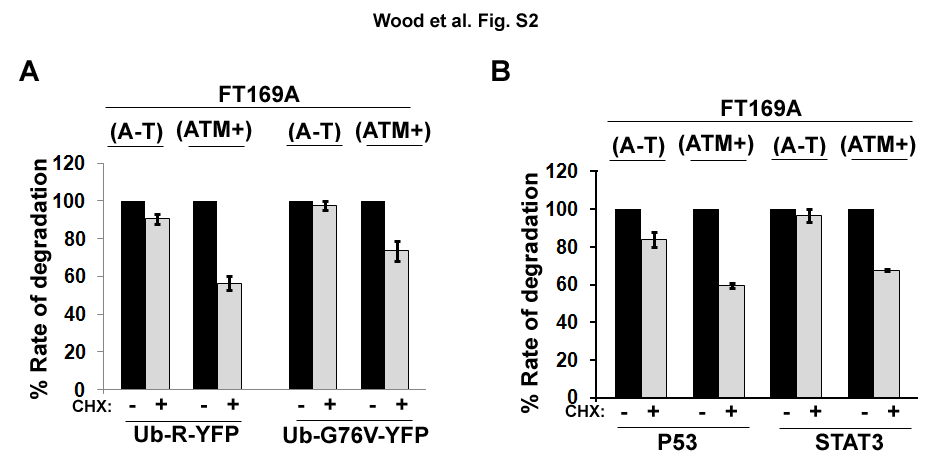

Supplement: Figure S2 — The 26S proteasome-mediated turnover of proteins is impaired in A-T cells. A. FT169A (A-T) and FT169A (ATM+) cells transfected with fluorescent reporter proteasome substrates (the ubiquitin fusion degradation substrate, UbG76V -YFP, and the N-end rule substrate, ubiquitin-arginine-YFP (Ub-R-YFP) for 12 hours. Proteasome inhibitor MG132 (0.5 µM) was then added to the transfection medium and cells were allowed to grow for an additional 12 hours. After washing (to remove MG132), cells were treated with protein synthesis inhibitor CHX (10 µg/ml) for three hours. The fluorescent reporter levels were detected with GFP antibodies. Average rate of degradation of Ub-G76V-YFP and Ub-R-YFP proteins (error bar represents S.E.M.) in FT169A (A-T) and FT169A (ATM+) cells measured using the Kodak image station 2000R from three independent experiments is shown in the bar graph. B. FT169A (A-T) and FT169A (ATM+) cells were treated with the protein synthesis inhibitor CHX (10 µg/ml) for 6 hours. Cell lysates were analyzed by immunoblotting using an anti-p53 and/or STAT3 antibody. Average rate of degradation of p53 and STAT3 proteins (error bar represents S.E.M.) in FT169A (A-T) and FT169A (ATM+) cells measured using the Kodak image station 2000R from three independent experiments is shown in the bar graph. (TIF) [file pone.0016422.s002.tif]

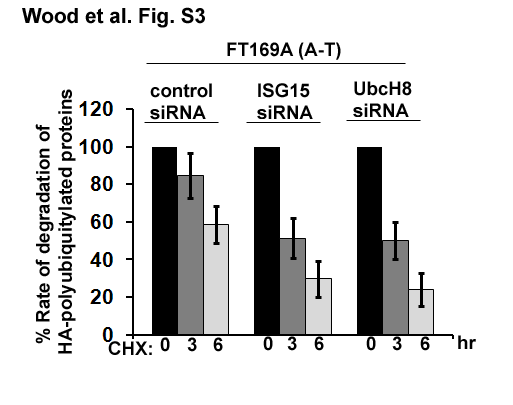

Supplement: Figure S3 — siRNA-mediated knockdown of ISG15 and UbcH8 increases degradation of polyubiquitylated proteins in A-T cells. FT169A (A-T) cells were transfected with ISG15 or UbcH8 siRNA for 72 hours. Cells were then treated with the protein synthesis inhibitor CHX (10 µg/ml) for 3 and 6 hours. Cell lysates were then analyzed by immunoblotting using anti-HA antibodies. Average rate of degradation of HA-polyubiquitylated proteins (error bar represents S.E.M.) in ISG15 or UbcH8 siRNA treated FT169A (A-T) cells measured using the Kodak image station 2000R from three independent experiments is shown in the bar graph. (TIF) [file pone.0016422.s003.tif]
